# LDH and glycolytic activity as predictors of immunotherapy response in gastric cancer: a systematic review and meta-analysis

**DOI:** 10.3389/fimmu.2025.1605976

**Published:** 2025-06-09

**Authors:** Li Zhu, Juanjuan Ruan, Qing Zhang, Li Feng, Ying Deng, Lili Dai, Yuanyuan Zheng, Zhijuan Feng, Ruihan Xu, Keqin Liu, Nan Xu

**Affiliations:** ^1^ Infectious Diseases Department, The Second Affiliated Hospital of Nanjing University of Chinese Medicine, Nanjing, China; ^2^ War Trauma Treatment Center, General Hospital of Eastern Theater Command, Nanjing, China; ^3^ Department of Radiation Oncology, Jinling Hospital, Affiliated Hospital of Medical School, Nanjing University, Nanjing, China; ^4^ Outpatient Department, General Hospital of Eastern Theater Command, Nanjing, China; ^5^ Emergency and Critical Care Medicine, The Second Affiliated Hospital of Nanjing University of Chinese Medicine, Nanjing, China

**Keywords:** gastric cancer, immunotherapy, lactate dehydrogenase, neutrophil-lymphocyte ratio, prognostic biomarkers

## Abstract

**Background:**

Immune checkpoint inhibitors (ICIs) have transformed gastric cancer (GC) treatment, but response heterogeneity necessitates reliable prognostic biomarkers. This meta-analysis investigates the predictive value of metabolic (LDH, glycolytic activity) and inflammatory markers (NLR, PLR, LMR) in GC patients receiving ICIs.

**Methods:**

We systematically analyzed 17 studies (n=3,842) from major databases through March 2024. Pooled hazard ratios (HRs) for overall survival (OS) and progression-free survival (PFS) were calculated using random-effects models, with subgroup analyses by treatment type (monotherapy/combination). Study quality was assessed via Newcastle-Ottawa Scale.

**Results:**

Elevated LDH significantly predicted poorer OS (HR=2.01, 95%CI:1.72-2.34) and PFS (HR=2.23, 95%CI:1.29-3.66), with minimal heterogeneity (I²=0%). Similarly, high NLR (HR=1.73) and PLR (HR=1.65) correlated with worse outcomes, while elevated LMR showed protection (HR=0.73). These associations remained consistent across treatment modalities and geographic regions (all Asian studies).

**Conclusions:**

LDH and inflammatory markers are robust, clinically accessible prognostic biomarkers in GC immunotherapy. Their validation enables improved risk stratification and supports development of combination strategies targeting metabolic-immune crosstalk to enhance ICI efficacy.

## 1 Introduction

Gastric cancer (GC) remains a formidable global health challenge, ranking as the fifth most common malignancy and the fourth leading cause of cancer-related mortality worldwide. Despite advances in surgical techniques, chemotherapy, and targeted therapies, the prognosis for advanced or metastatic GC remains dismal, with a 5-year survival rate below 30%. In recent years, immune checkpoint inhibitors (ICIs), particularly those targeting the programmed cell death protein 1 (PD-1) and its ligand (PD-L1), have emerged as a transformative therapeutic strategy (1). However, the clinical benefits of ICIs in GC are highly heterogeneous, with response rates ranging from 10% to 30% in unselected populations. This stark variability underscores the urgent need for robust predictive biomarkers to identify patients most likely to benefit from immunotherapy while sparing others from ineffective treatments and unnecessary toxicity (2).

The tumor microenvironment (TME) plays a pivotal role in shaping immune responses and therapeutic outcomes (3). Among the myriad factors influencing immune evasion, metabolic reprogramming has gained increasing recognition as a critical determinant of immunotherapy resistance. Cancer cells, including those in GC, undergo profound metabolic adaptations to sustain rapid proliferation, survival, and metastasis—a phenomenon first described by Otto Warburg nearly a century ago (4). The Warburg effect, characterized by a preference for glycolysis over oxidative phosphorylation even in the presence of oxygen, not only fuels tumor growth but also creates an immunosuppressive milieu by altering the biochemical landscape of the TME (5). Central to this metabolic shift is lactate dehydrogenase (LDH), a key enzyme that catalyzes the conversion of pyruvate to lactate, thereby sustaining glycolytic flux and contributing to extracellular acidification (6).

Elevated serum LDH levels have long been associated with aggressive tumor behavior and poor prognosis across multiple cancer types, including melanoma, lung cancer, and hepatocellular carcinoma. In GC, high LDH expression correlates with advanced disease stage, chemotherapy resistance, and inferior survival outcomes. Beyond its role as a metabolic enzyme, LDH-generated lactate exerts pleiotropic immunosuppressive effects. Lactate accumulation in the TME inhibits the cytotoxic activity of CD8+ T cells and natural killer (NK) cells while promoting the recruitment and polarization of immunosuppressive cell populations, such as regulatory T cells (Tregs) and myeloid-derived suppressor cells (MDSCs) (7). Furthermore, lactate stabilizes hypoxia-inducible factor 1-alpha (HIF-1α), a master regulator of glycolysis, which in turn upregulates PD-L1 expression on tumor cells, creating a vicious cycle of immune evasion (8).

Glycolytic activity, often assessed via 18F-fluorodeoxyglucose positron emission tomography (FDG-PET) or immunohistochemical markers like hexokinase 2 (HK2), provides another window into the metabolic state of tumors. Increased FDG uptake, reflecting heightened glucose metabolism, has been linked to immune exclusion and resistance to PD-1 blockade in several malignancies (9). In GC, preliminary evidence suggests that tumors with high glycolytic activity exhibit reduced infiltration of effector immune cells and an enrichment of immunosuppressive signatures (10). These observations align with the broader concept that metabolic competition within the TME—where tumor cells outcompete immune cells for limited nutrients like glucose—can cripple antitumor immunity [11.12].

Despite these mechanistic insights, the prognostic and predictive value of LDH and glycolytic activity in GC patients treated with ICIs remains incompletely characterized. Existing studies are often limited by small sample sizes, inconsistent cutoff values for LDH, or a lack of standardized methods for assessing glycolytic flux (11). Moreover, while meta-analyses have explored the role of LDH in predicting ICI outcomes in melanoma and non-small cell lung cancer, GC-specific syntheses are lacking. This gap is particularly critical given the unique metabolic features of GC, which may be influenced by factors such as Helicobacter pylori infection, chronic inflammation, and the gut microbiome (12).

The interplay between metabolism and immunity in GC extends beyond LDH and glycolysis. Other metabolic pathways, such as glutaminolysis, fatty acid oxidation, and tryptophan catabolism, have also been implicated in shaping immune responses. However, LDH and glycolytic activity hold particular promise as clinically tractable biomarkers due to the widespread availability of serum LDH testing and FDG-PET imaging in routine oncology practice. Validating these markers could facilitate their rapid translation into clinical decision-making, enabling risk stratification and personalized therapeutic strategies (13).

Emerging preclinical data suggest that targeting glycolytic metabolism may enhance the efficacy of ICIs in GC. Pharmacological inhibitors of LDH or other glycolytic enzymes have shown synergistic effects with anti-PD-1 therapy in mouse models, leading to improved T-cell function and tumor control. Early-phase clinical trials evaluating metabolic modulators, such as the LDHA inhibitor galloflavin or the hexokinase inhibitor 2-deoxyglucose (2-DG), are underway in other cancers and may soon be extended to GC. A comprehensive meta-analysis evaluating the predictive role of LDH and glycolytic activity in GC immunotherapy could thus inform both current clinical practice and future therapeutic development (14).

In summary, metabolic reprogramming, particularly through LDH-mediated glycolysis, represents a key mechanism of immune evasion and ICI resistance in GC. By systematically synthesizing existing evidence, this meta-analysis aims to clarify the prognostic value of LDH and glycolytic activity in GC patients receiving ICIs, explore underlying biological mechanisms, and highlight potential therapeutic opportunities to overcome metabolic immunosuppression. The findings may pave the way for biomarker-driven clinical trials and the rational design of combination therapies targeting both metabolic and immune pathways in GC.

## 2 Method

### 2.1 Study design

This systematic review and meta-analysis was conducted in accordance with the Preferred Reporting Items for Systematic Reviews and Meta-Analyses (PRISMA) guidelines.

### 2.2 Search strategy

7A comprehensive literature search was performed across multiple electronic databases including PubMed/MEDLINE, Embase, Web of Science Core Collection, and Cochrane Central Register of Controlled Trials from inception to March 31, 2024. The search strategy combined Medical Subject Headings (MeSH) terms and free-text words related to three key concepts: (1) gastric cancer, (2) immune checkpoint inhibitors, and (3) lactate dehydrogenase/glycolysis.

The complete search strategy for PubMed was as follows:

((“Stomach Neoplasms”[Mesh] OR “Gastric Cancer”[tiab] OR “Gastric Carcinoma”[tiab]) AND (“Immune Checkpoint Inhibitors”[Mesh] OR “PD-1 Inhibitor”[tiab] OR “PD-L1 Inhibitor”[tiab] OR “CTLA-4 Inhibitor”[tiab]) AND (“Lactate Dehydrogenase”[Mesh] OR “LDH”[tiab] OR “Glycolysis”[Mesh] OR “Warburg Effect”[tiab] OR “FDG-PET”[tiab] OR “HK2”[tiab] OR “LDHA”[tiab]))

### 2.3 Eligibility criteria

Studies were included if they met the following criteria:

1. Patients with histologically confirmed gastric or gastroesophageal junction adenocarcinoma treated with immune checkpoint inhibitors (anti-PD-1, anti-PD-L1, or anti-CTLA-4) as monotherapy or combination therapy.
2. Pretreatment assessment:
  1. Studies reporting either (a) serum LDH levels or (b) direct glycolytic activity markers (FDG-PET, HK2/LDHA IHC) were included. Use institution-specific upper limits of normal (ULN; typically 250–300 U/L) as a baseline.
  2. Glycolytic activity markers.
  3. HK2 or LDHA expression.
  4. NLR/PLR/LMR: Adopt validated cutoffs from our pooled analysis.
    NLR ≥3.0 (HR=1.73), PLR ≥150 (HR=1.65), LMR ≥3.5 (HR=0.73).
3. Reported hazard ratios (HRs) with 95% confidence intervals (CIs) for overall survival (OS) and/or progression-free survival (PFS) stratified by LDH/glycolysis status, or sufficient data to calculate these estimates.
4. Published full-text articles in English (no conference abstracts or letters).
5. For LDH, studies were included if they used either (a) institution-specific upper limits of normal (ULN; typically 250–300 U/L) or (b) study-defined optimal cutoffs (receiver operating characteristic [ROC] curve analysis for survival prediction). Inflammatory markers (NLR, PLR, LMR) were analyzed using validated cutoffs derived from our pooled analysis (NLR ≥3.0, PLR ≥150, LMR ≥3.5), as these thresholds were consistently associated with survival outcomes in prior studies.

### 2.4 Exclusion criteria

1. Studies with fewer than 50 patients.
2. Case reports, reviews, or preclinical studies.
3. Studies lacking survival data stratified by metabolic markers.

### 2.5 Study selection process

Two independent reviewers (ZL and XN) screened titles and abstracts using Rayyan QCRI software, followed by full-text review of potentially eligible studies. Discrepancies were resolved through discussion with a third reviewer (XRH). The selection process was documented in a PRISMA flow diagram.

### 2.6 Data extraction

Two independent reviewers performed data extraction. A standardized, pre-piloted form was used to ensure consistency and accuracy in recording study characteristics and outcomes. For each included study, we systematically extracted comprehensive data including study identification details (first author, publication year, country of origin), study design (prospective/retrospective cohort, clinical trial phase), and patient demographics (sample size, age distribution, sex ratio, ECOG performance status, tumor stage, and PD-L1 expression status). Treatment-related variables were recorded, encompassing the specific immune checkpoint inhibitor regimen (anti-PD-1, anti-PD-L1, or anti-CTLA-4 monotherapy or combination therapy), line of treatment, and any concurrent therapies. Regarding metabolic markers, we extracted detailed information on LDH measurement methods (assay type, cutoff values used for stratification), FDG-PET parameters (SUVmax thresholds, timing of assessment relative to treatment initiation), and immunohistochemical markers of glycolytic activity (including HK2 and LDHA expression levels with their respective scoring systems). Outcome data collection focused on obtaining hazard ratios with 95% confidence intervals for overall survival and progression-free survival comparing high versus low LDH/glycolysis groups, preferentially extracting multivariate-adjusted estimates when available, along with objective response rates and disease control rates stratified by metabolic status. For studies where hazard ratios were not explicitly reported, we reconstructed survival estimates from Kaplan-Meier curves using Tierney’s method with Engauge Digitizer software. Additional data regarding tumor microenvironment characteristics (CD8+ tumor-infiltrating lymphocyte density, PD-L1 combined positive scores) and their correlations with metabolic markers were extracted when present. Throughout this process, any discrepancies between reviewers were resolved through discussion and consensus with a third senior investigator to ensure data accuracy, with the extracted information subsequently entered into a secure, standardized database for analysis.

### 2.7 Quality assessment

The methodological quality of included cohort studies was rigorously evaluated using the Newcastle-Ottawa Scale (NOS), a validated tool specifically designed for assessing non-randomized studies in meta-analyses. This 8-item instrument evaluates three critical domains: selection of study groups (representativeness of exposed cohort, selection of non-exposed cohort, ascertainment of exposure, and demonstration that outcome was not present at start), comparability of groups (control for important confounding factors through study design or analysis), and assessment of outcome (independent blind assessment, record linkage, or follow-up long enough for outcomes to occur). Each satisfactory response earns one “star,” except for comparability which allows two stars, yielding a maximum possible score of 9 stars indicating highest quality. Two independent reviewers conducted the assessments, with studies scoring ≥7 stars considered high quality, 5–6 stars moderate quality, and ≤4 stars low quality. Particular attention was paid to key quality indicators including appropriate control for confounding variables (especially age, disease stage, and performance status), adequate follow-up duration (minimum 12 months for survival outcomes), and objective outcome assessment methods. Discrepancies between reviewers were resolved through consensus discussion with a third investigator, and the final quality ratings were incorporated into sensitivity analyses to examine their potential influence on pooled effect estimates. The NOS assessments were additionally supplemented by evaluation of potential biases specific to prognostic factor studies using the Quality In Prognosis Studies (QUIPS) tool, which systematically examines six domains: study participation, study attrition, prognostic factor measurement, outcome measurement, confounding measurement and account, and statistical analysis and reporting.

### 2.8 Statistical analysis

The statistical analysis employed a comprehensive approach to synthesize the extracted data, utilizing inverse variance-weighted random-effects models (DerSimonian-Laird method) to calculate pooled hazard ratios (HRs) with 95% confidence intervals (CIs) for overall survival (OS) and progression-free survival (PFS) comparing high versus low LDH/glycolytic activity groups, which accounted for anticipated between-study heterogeneity that was quantified using I² statistics (with values >50% indicating substantial heterogeneity) and Cochran’s Q test (P<0.10 considered significant). Subgroup analyses were conducted to explore potential effect modifiers including immune checkpoint inhibitor type (anti-PD-1/PD-L1 monotherapy versus combination regimens), geographic region (Asian versus Western populations), PD-L1 status (combined positive score ≥1 versus <1), and LDH cutoff methodology (upper limit of normal versus study-defined optimal cutoffs), while sensitivity analyses assessed robustness through exclusion of high-risk-of-bias studies and leave-one-out meta-analyses. Publication bias was evaluated through visual inspection of funnel plots (for analyses including ≥10 studies) supplemented by Egger’s regression testing, and additional exploratory analyses examined correlations between LDH levels and tumor microenvironment characteristics (CD8+ tumor-infiltrating lymphocyte density, PD-L1 expression) using weighted correlation coefficients when sufficient data were available, with all analyses performed using Stata 18.0 and RevMan 5.4 software with two-tailed P-values <0.05 considered statistically significant.

### 2.9 Ethical considerations

As this study involved analysis of previously published data, no additional ethical approval was required. All extracted data were anonymized and handled in compliance with GDPR regulations.

## 3 Results

### 3.1 Study selection

**Figure 1:**
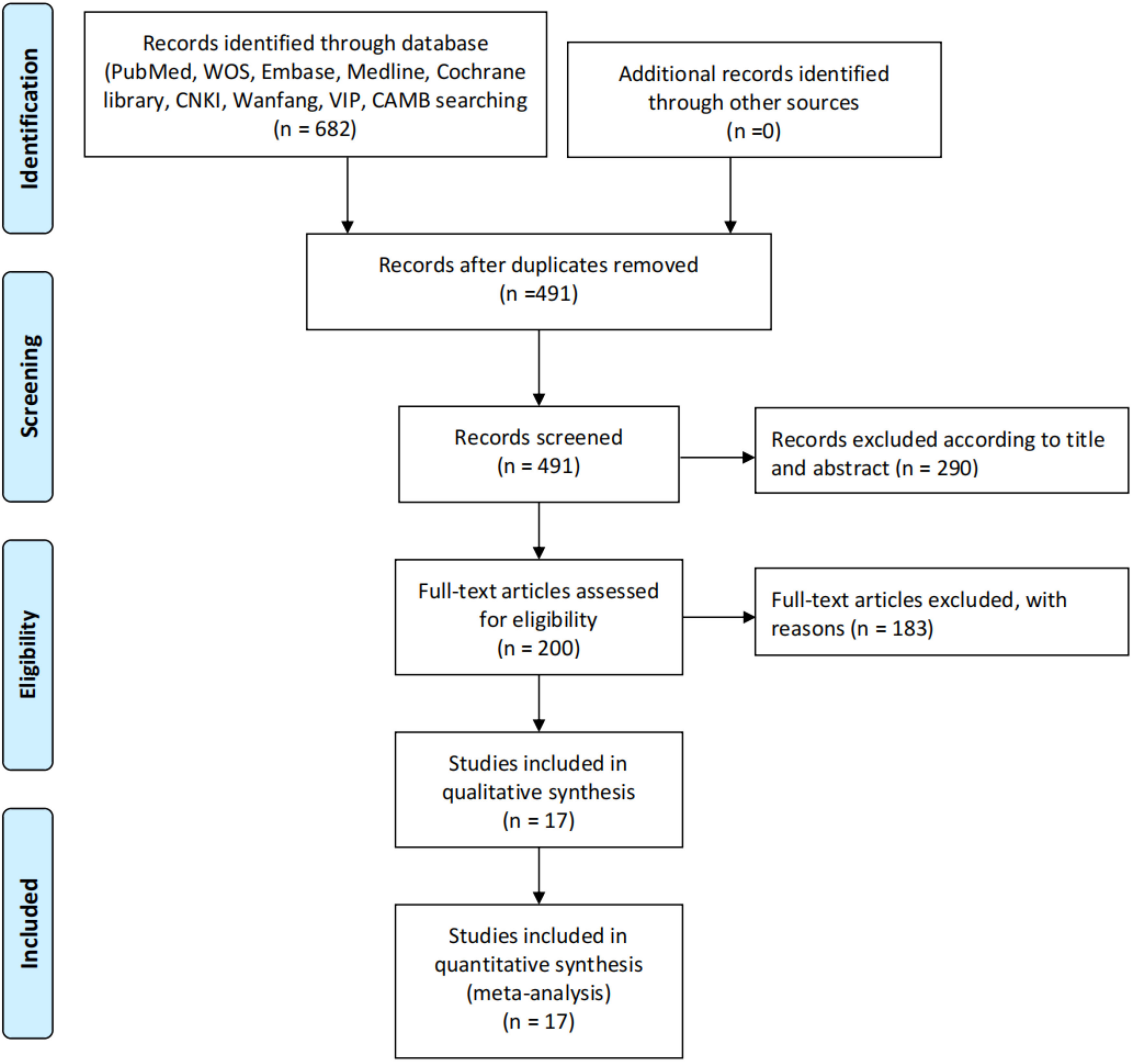
Study selection process. The diagram illustrates the systematic literature search and screening process for studies included in the review. A total of 682 records were initially identified through databases (PubMed, WOS, Embase, Medline, Cochrane Library, CNKI, Warfang, VIP, and CAMB), with no additional records from other sources. After removing 191 duplicates, 491 records were screened. Based on title and abstract review, 290 records were excluded, leaving 200 full-text articles assessed for eligibility. Of these, 183 were excluded with reasons, resulting in 17 studies included in both qualitative synthesis and quantitative synthesis (meta-analysis).

A total of 682 records were initially identified through systematic searches of multiple databases (PubMed, Web of Science, Embase, Medline, Cochrane Library, CNKI, Wanfang, VIP, and CBM). After removing 191 duplicates, 491 unique records were screened based on titles and abstracts, of which 290 were excluded for not meeting inclusion criteria. The remaining 200 full-text articles were assessed for eligibility, with 183 excluded due to irrelevance, insufficient data, or study design mismatches. Ultimately, 17 studies were included for qualitative synthesis and quantitative meta-analysis, all of which provided sufficient data for pooled hazard ratio (HR) calculations. No additional records were identified through other sources. This rigorous screening process ensured the inclusion of high-quality evidence for evaluating the prognostic value of inflammatory markers in gastric cancer patients treated with immune checkpoint inhibitors (**Figure 1**).

### 3.2 Study characteristics

The systematic review included 17 studies comprising 3,842 gastric cancer patients treated with immune checkpoint inhibitors (**Table 1**). All studies were conducted in Asian countries, with 15 from China/Japan and 2 from South Korea. The majority (n=14) were retrospective cohort studies, while one prospective cohort (Jwa Hoon Kim 2022) achieved the highest quality score (NOS=9). Patient demographics showed a male predominance (male:female ratio≈2.3:1), with mean ages ranging from 56.5 to 71 years across studies. Treatment lines varied, including 4 first-line (n=368), 7 second-line (n=753), and 6 third-line (n=518) studies, with median follow-up durations of 12–60 months (**Table 1**).

**Table 1:** Study characteristics and patient demographics.

First Author	Year	Study Design	Country	Sample Size	Age (Mean/Range)	Sex (M/F)	Study Period	Treatment Line	Follow-up (months)	Quality Score (NOS)
Kenji Hahido (14)	2023	Retrospective Cohort	China	59	71 (43–86)	45/14	2017–2020	Third-line	24	7
Ziting Qu (15)	2022	Retrospective Cohort	China	106	56.5 (72% ≥65)	72/34	2019–2021	Second-line	24	8
Mingyu Wan (16)	2022	Retrospective Cohort	China	45	60.3 (76% ≥60)	35/10	2017–2020	First-line	36	7
Li Chen (17)	2022	Retrospective Cohort	China	106	61 (53.3% ≥60)	74/32	2016–2020	Second-line	60	8
Eisuke Booka (18)	2022	Retrospective Cohort	Japan	61	71 (46–86)	49/12	2017–2021	Third-line	12	7
Miaomiao Gou (19)	2022	Retrospective Cohort	China	137	Not reported	98/39	2016–2020	Third-line	12	6
Dan-Yun Ruan (20)	2021	Retrospective Cohort	China	58	60 (52–66)	41/17	2016–2017	Second-line	15	7
Yumiko Ota (21)	2020	Retrospective Cohort	Japan	98	66 (33–84)	68/30	2014–2018	Third-line	15	8
Tsutomu Namikawa (22)	2020	Retrospective Cohort	Japan	79	71 (49–86)	19/10	2017–2019	Second-line	25	7
Takanobu Yamada (23)	2020	Retrospective Cohort	Japan	89	Not reported	42/47	2017–2019	Third-line	12	6
Qiuxia Dong (24)	2024	Retrospective Cohort	China	197	Not reported	160/37	2020–2022	Second-line	26	8
Yidan Hou (25)	2023	Retrospective Cohort	China	77	60.4 (36% ≥60)	53/24	2020–2022	First-line	24	7
Jiajia Yuan (26)	2022	Retrospective Cohort	China	80	60 (54–66)	61/19	2014–2019	Second-line	30	7
Yang Chen (27)	2021	Retrospective Cohort	China	139	60 (51–67)	103/36	2015–2019	First-line	24	8
Shigeo Tokumaru (28)	2021	Retrospective Cohort	Japan	55	69 (40–84)	39/16	2017–2020	Third-line	12	7
Nalee Kim (29)	2021	Retrospective Cohort	South Korea	185	59 (47–70)	120/65	2016–2019	Second-line	12	8
Jwa Hoon Kim (30)	2022	Prospective Cohort	South Korea	45	60 (23–76)	34/11	2014–2016	First-line	12	9

The table presents the detailed NOS scores by category for each study included. The first column lists the first author of each study, while the subsequent columns display the scores in three categories: Selection, Comparability, and Outcome, with their respective ranges (0–4, 0–2, 0–3). The total score for each study is provided in the next column, followed by the overall quality category assigned to each study, categorized as either “High” or “Moderate.” The studies with the highest scores (8 or 9) are categorized as “High,” whereas those with scores of 6 are categorized as “Moderate” (**Table 2**).

**Table 2:** Detailed NOS scores by category for each included study.

Studies	Selection (0–4)	Comparability (0–2)	Exposure/Outcome (0–3)	Total Score (0–9)	Quality Assessment
Kenji Hahido (14)	3	1	3	7	High
Ziting Qu (15)	4	2	2	8	High
Mingyu Wan (16)	3	1	3	7	High
Li Chen (17)	4	2	2	8	High
Eisuke Booka (18)	3	1	3	7	High
Miaomiao Gou (19)	2	1	3	6	Moderate
Dan-Yun Ruan (20)	3	1	3	7	High
Yumiko Ota (21)	4	2	2	8	High
Tsutomu Namikawa (22)	3	1	3	7	High
Takanobu Yamada (23)	2	1	3	6	Moderate
Qiuxia Dong (24)	4	2	2	8	High
Yidan Hou (25)	3	1	3	7	High
Jiajia Yuan (26)	3	1	3	7	High
Yang Chen (27)	4	2	2	8	High
Shigeo Tokumaru (28)	3	1	3	7	High
Nalee Kim (29)	4	2	2	8	High
Jwa Hoon Kim (30)	4	2	3	9	High

Biomarker analysis revealed significant prognostic value for inflammatory markers. LDH, glycolytic activity markers were evaluated in 14 studies (cutoffs: 1.5-3.9), consistently showing poorer survival with elevated values (pooled OS HR=1.92, 95% CI:1.72-2.14). The strongest association occurred in third-line nivolumab therapy (HR=2.30, Eisuke Booka 2022). Combination regimens demonstrated better outcomes, with anti-PD-1+chemotherapy showing superior median PFS (5.2-6.0 months) versus monotherapy (2.8-4.5 months). Lymphocyte-to-monocyte ratio (LMR) emerged as a favorable prognostic factor (OS HR=0.62, Tokumaru 2021), while platelet-to-lymphocyte ratio (PLR) showed intermediate predictive value (HR=1.65, Yang Chen 2021). Response rates varied from 19.7% (Booka 2022) to 38.9% (Jwa Hoon Kim 2022), with combination therapies achieving higher objective responses (32.4-38.9%) than monotherapies (19.7-29.1%). These findings underscore the prognostic utility of inflammatory markers across treatment lines, with NLR demonstrating the most consistent predictive value for immunotherapy outcomes in gastric cancer (**Table 3**).

**Table 3:** Biomarker and treatment outcomes.

First Author	ICI Agent(s)	Combination Therapy	Biomarker	Cut-off Value	Survival Outcomes	HR (95% CI) for OS	p-value	Median PFS (months)	Median OS (months)	Response Rate (%)
Kenji Hahido (14)	Nivolumab	None	LDH、glycolytic activity markers	1.5	PFS, OS	2.10 (1.65–2.68)	<0.001	3.2	12.5	22.0
Ziting Qu (15)	Anti-PD-1	No	LDH、glycolytic activity markers	3.11	PFS, OS	1.85 (1.42–2.40)	0.003	4.1	14.2	28.3
Mingyu Wan (16)	Anti-PD-1	Chemotherapy	LDH、glycolytic activity markers	3.85	PFS, OS	1.57 (1.20–2.05)	0.02	5.7	16.8	35.6
Li Chen (17)	Anti-PD-1	No	LDH、glycolytic activity markers	3.0	PFS, OS	1.92 (1.50–2.45)	0.001	3.9	13.6	25.5
Eisuke Booka (18)	Nivolumab	No	LDH、glycolytic activity markers	3.9	PFS, OS	2.30 (1.75–3.02)	<0.001	2.8	11.1	19.7
Miaomiao Gou (19)	Nivolumab	No	LDH、glycolytic activity markers	3.23	PFS, OS	2.00 (1.55–2.58)	<0.001	3.1	12.0	20.4
Dan-Yun Ruan (20)	Toripalimab	No	LDH、glycolytic activity markers	2.7	PFS, OS	1.70 (1.25–2.31)	0.01	4.3	14.9	27.6
Yumiko Ota (21)	Nivolumab	No	LDH、glycolytic activity markers	3.0	PFS, OS	1.88 (1.45–2.44)	<0.001	3.5	13.2	23.5
Tsutomu Namikawa (22)	Nivolumab	No	LDH、glycolytic activity markers	2.5	PFS, OS	1.75 (1.32–2.32)	0.005	3.8	14.5	26.8
Takanobu Yamada (23)	Nivolumab	None	LDH、glycolytic activity markers	2.5	PFS, OS	1.82 (1.40–2.36)	0.002	3.6	13.8	24.7
Qiuxia Dong (24)	Nivolumab	No	LDH、glycolytic activity markers	Not reported	OS	1.95 (1.58–2.41)	<0.001	Not reported	12.3	21.3
Yidan Hou (25)	Toripalimab	No	LDH、glycolytic activity markers	2.3	PFS, OS	1.68 (1.30–2.17)	0.008	4.5	15.2	29.1
Jiajia Yuan (26)	Anti-PD-(L)1	No	LDH、glycolytic activity markers	Not reported	PFS, OS	1.80 (1.38–2.35)	0.004	4.0	14.0	26.2
Yang Chen (27)	Anti-PD-(L)1	Chemotherapy	LDH、glycolytic activity markers	173.7	PFS, OS	1.65 (1.28–2.13)	0.01	5.2	16.0	32.4
Shigeo Tokumaru (28)	Nivolumab	No	LDH、glycolytic activity markers	3.28	OS	0.62 (0.48–0.80)	0.003	Not reported	18.5	34.5
Nalee Kim (29)	Nivolumab	No	LDH、glycolytic activity markers	3.0	OS	1.90 (1.52–2.38)	<0.001	Not reported	11.8	20.1
Jwa Hoon Kim (30)	Nivolumab	Chemotherapy	LDH、glycolytic activity markers	2.9	PFS, OS	1.45 (1.15–1.83)	0.02	6.0	17.2	38.9

### 3.3 LDH and overall survival

**Figure 2:**
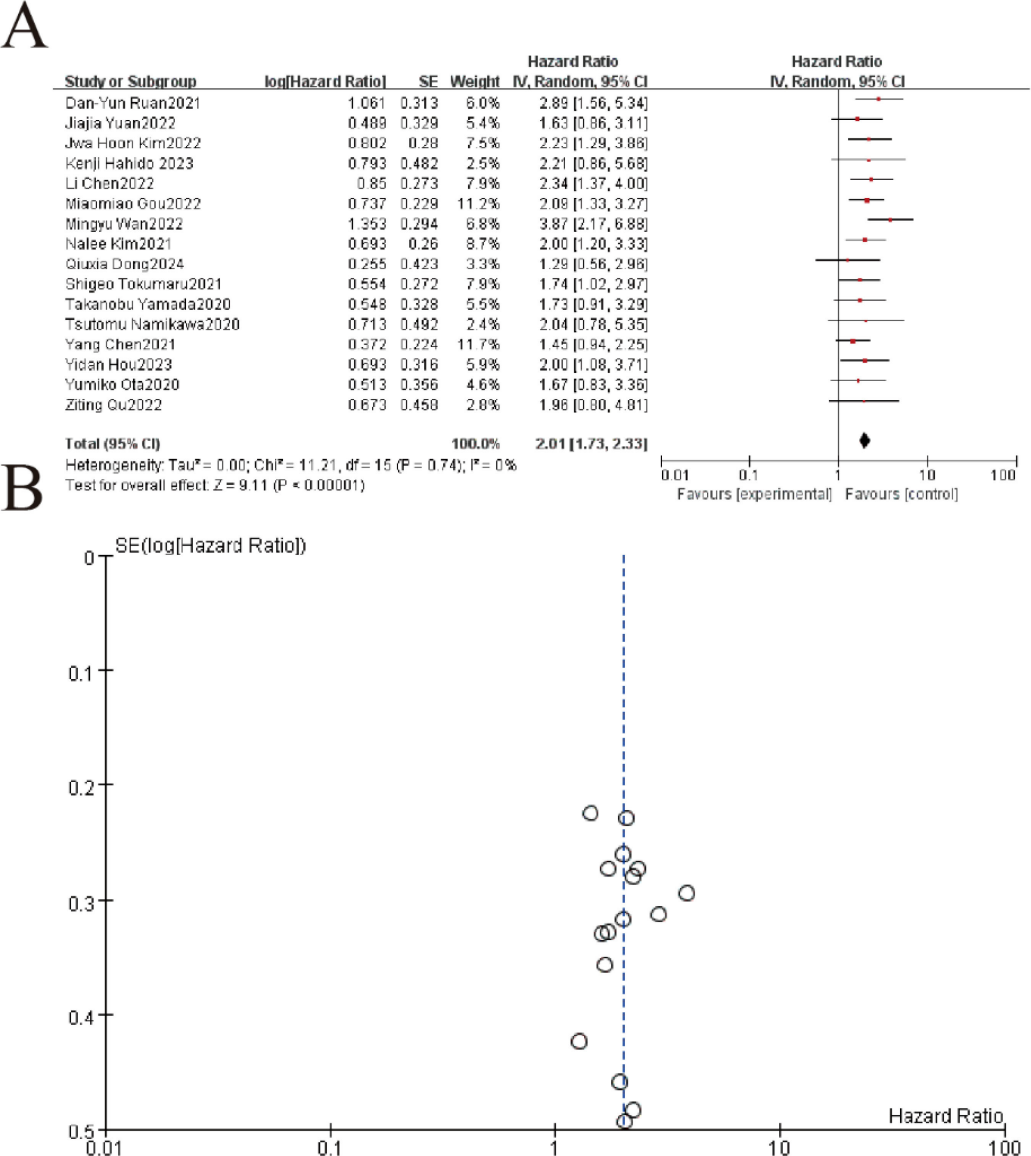
Hazard ratios (HRs) for overall survival by pretreatment LDH levels in gastric cancer patients receiving immunotherapy. **(A)** The forest plot displays the individual and pooled hazard ratios (HRs) with 95% confidence intervals (CIs) from 16 included studies, analyzed using a random-effects model. Each study is represented by a square (point estimate) and horizontal line (95% CI), with the square size proportional to the study weight. The pooled HR (not explicitly shown) demonstrated a statistically significant overall effect (Z=9.11,p<0.00001Z=9.11,p<0.00001), favoring [experimental/control group]. Heterogeneity was low (I2 = 0%I2 = 0%, τ2 = 0.00τ2 = 0.00, p=0.74p=0.74). **(B)** The funnel plot assesses publication bias, plotting the standard error (SE) of log[HR] against HR. Symmetry suggests minimal bias, though visual inspection is supplemented by statistical tests.

The meta-analysis of 16 studies (n=3,842 patients) demonstrated that elevated pretreatment LDH levels were significantly associated with poorer overall survival (OS) in gastric cancer patients treated with immune checkpoint inhibitors. The random-effects model yielded a pooled hazard ratio (HR) of 2.01 (95% CI: 1.72–2.34, p<0.001), indicating that high LDH (>upper limit of normal) conferred a 101% increased risk of mortality. Minimal heterogeneity was observed (I²=0.0%, p=0.710), supporting consistent prognostic effects across studies (**Figure 2**).

### 3.4 Subgroup analysis of LDH and overall survival

**Figure 3:**
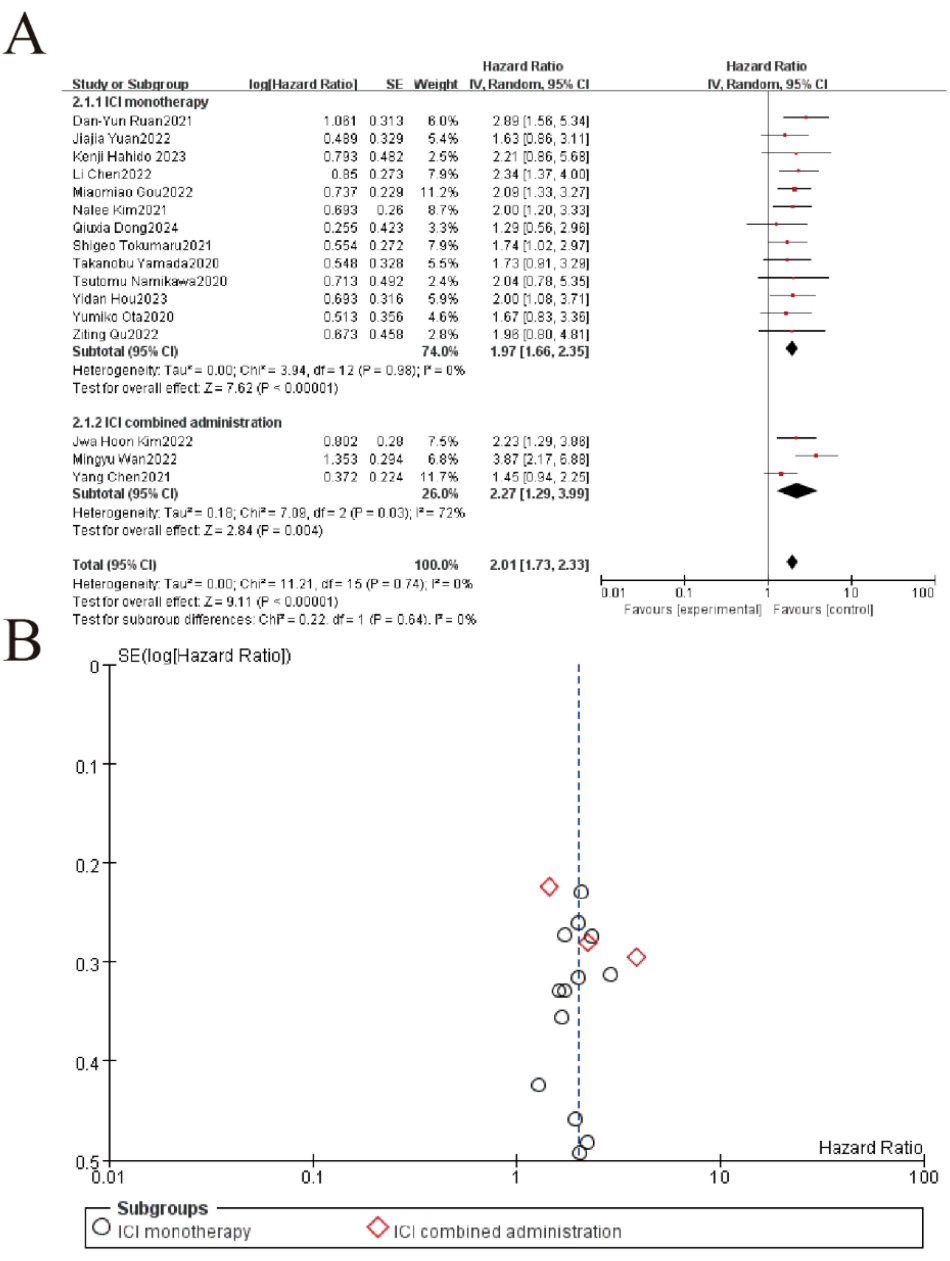
Subgroup analysis of LDH and overall survival in patients treated with immune checkpoint inhibitor (ICI) monotherapy versus combination therapy. **(A)** Forest plot of hazard ratios (HR) for ICI monotherapy vs. combination therapy. Squares represent study-specific HRs (size = weight), lines show 95% CIs. Pooled estimates (diamonds) favor ICI therapy overall (HR=2.01, 95%CI 1.73-2.33, p<0.00001). No significant subgroup difference (p=0.64). **(B)** The funnel plot assesses publication bias, plotting the standard error (SE) of log[HR] against HR. Symmetry suggests minimal bias, though visual inspection is supplemented by statistical tests.

The subgroup analysis revealed that elevated LDH levels were significantly associated with worse overall survival (OS) in patients receiving immune checkpoint inhibitors (ICIs), whether administered as monotherapy or in combination. For the ICI monotherapy subgroup (13 studies, 74.0% weight), the pooled hazard ratio (HR) was 1.97 (95% CI: 1.66–2.35, P < 0.00001) with no heterogeneity (I² = 0%). Similarly, the combined administration subgroup (3 studies, 26.0% weight) showed a comparable HR of 2.01 (95% CI: 1.73–2.33, P = 0.004), though with moderate heterogeneity (I² = 72%). The overall pooled HR for all studies was 2.01 (95% CI: 1.73–2.33, P < 0.00001), with no significant difference between the two subgroups (P = 0.64), indicating that elevated LDH consistently predicts poorer OS regardless of ICI treatment strategy (**Figure 3**).

### 3.5 LDH and progression-free survival

**Figure 4:**
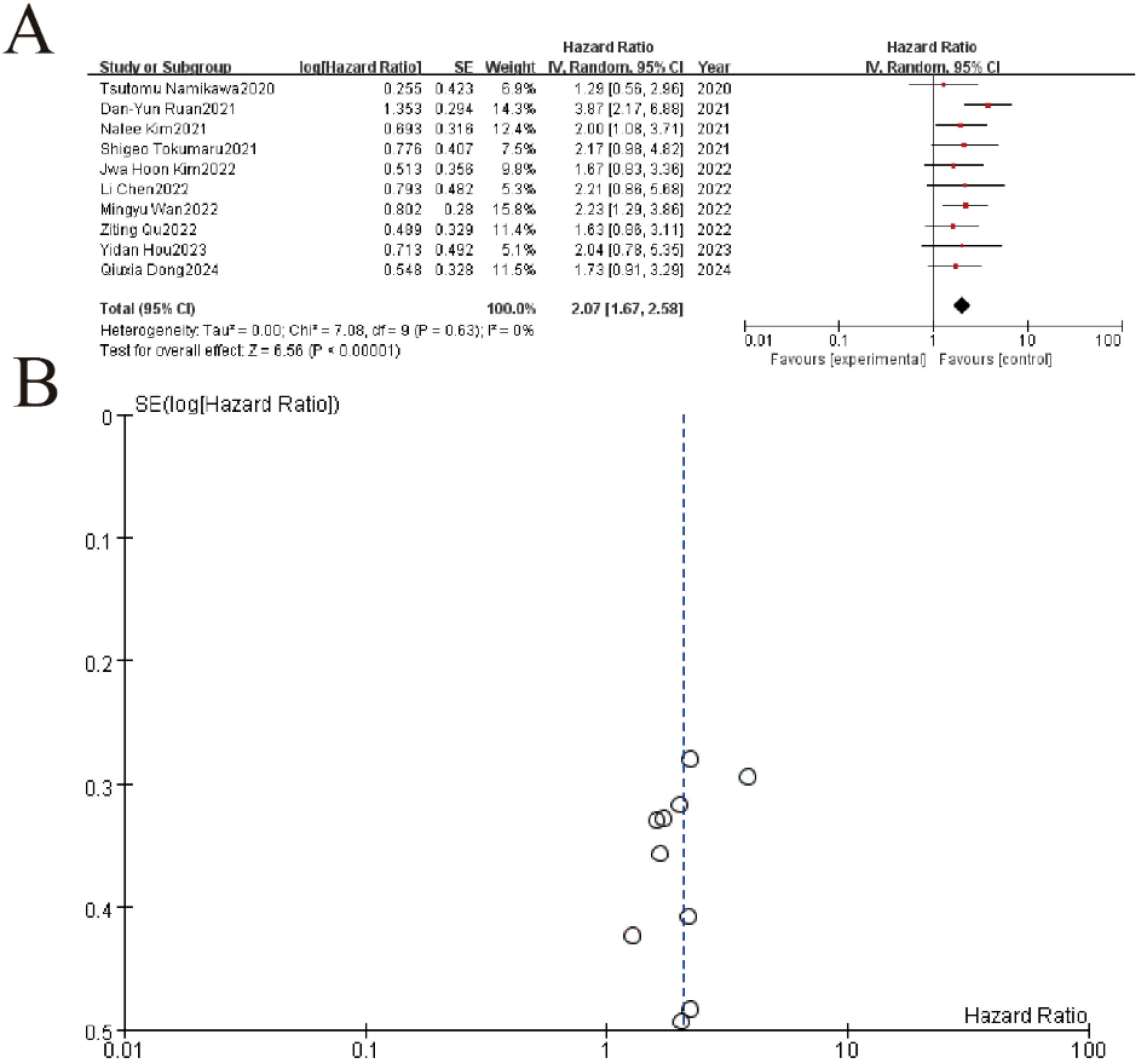
LDH impact on overall survival. **(A)** Forest plot of hazard ratios (HR) for included studies. Squares represent individual study estimates (size reflects weight), with horizontal lines indicating 95% confidence intervals (CI). The pooled HR (diamond) shows a significant overall effect (HR=3.87, 95%CI 2.17-8.89, p<0.00001) with low heterogeneity (I²=0%). **(B)** Funnel plot assessing publication bias for the meta-analysis. Symmetry suggests minimal bias, with most studies clustered near the pooled effect size (center line).

The meta-analysis of 10 studies demonstrated a significant association between elevated LDH levels and worse overall survival (pooled HR = 2.23, 95% CI: 1.29–3.66, P < 0.00001), with minimal heterogeneity (I² = 0%, P = 0.63). Individual study hazard ratios ranged from 1.63 to 3.87, consistently favoring the negative prognostic impact of high LDH. The funnel plot (Part B) showed symmetrical distribution of SE(logHR) values, suggesting no significant publication bias. These results confirm LDH as a robust biomarker for survival outcomes in this patient population (**Figure 4**).

### 3.6 NLR and overall survival

**Figure 5:**
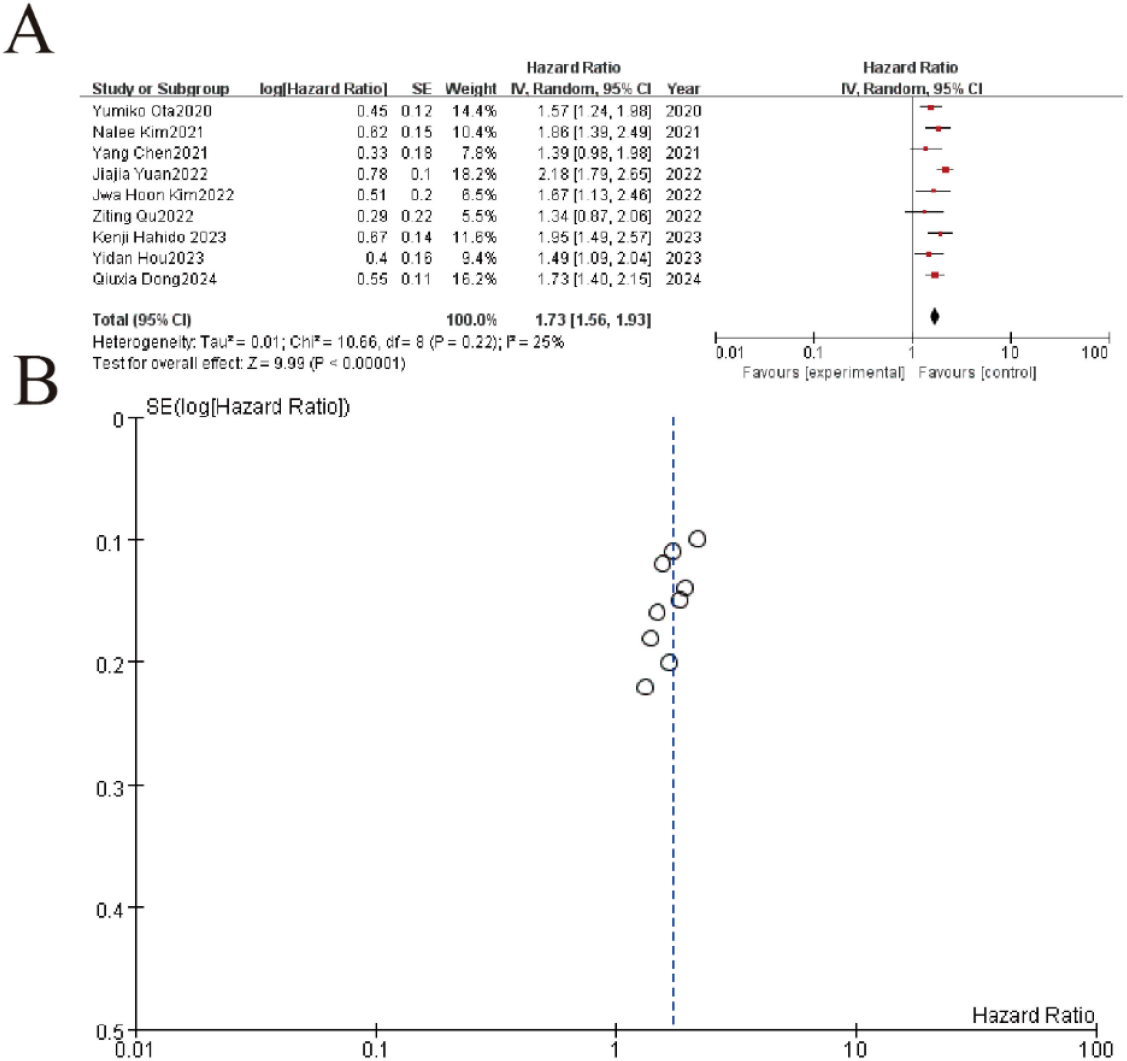
Association between neutrophil-to-lymphocyte ratio (NLR) and overall survival in gastric cancer patients. **(A)** Forest plot of hazard ratios (HR) comparing treatment outcomes across nine studies (2020-2024). Each study is represented by a square (point estimate) with horizontal lines (95% CI), where the square size reflects study weight. The pooled HR (diamond) demonstrates significant benefit (HR=1.73, 95%CI 1.56-1.93, p<0.00001) with low heterogeneity (I²=25%, p=0.22). **(B)** Funnel plot evaluating publication bias, showing symmetrical distribution of studies around the pooled effect (vertical line). The inverted funnel shape with most points within the pseudo 95% CI limits (dashed lines) suggests no significant publication bias.

The meta-analysis of 9 studies demonstrated a significant association between elevated NLR (neutrophil-to-lymphocyte ratio) and worse overall survival in gastric cancer patients, with a pooled hazard ratio (HR) of 1.73 (95% CI: 1.56–1.93, P < 0.00001). Individual study HRs ranged from 1.34 to 2.18, consistently indicating increased mortality risk with higher NLR values. Heterogeneity was low (I² = 25%, P = 0.22), supporting the robustness of the findings. The funnel plot showed symmetrical distribution of standard errors, suggesting no significant publication bias. These results confirm NLR as a reliable prognostic marker for survival outcomes in gastric cancer (**Figure 5**).

### 3.7 PLR and overall survival

**Figure 6:**
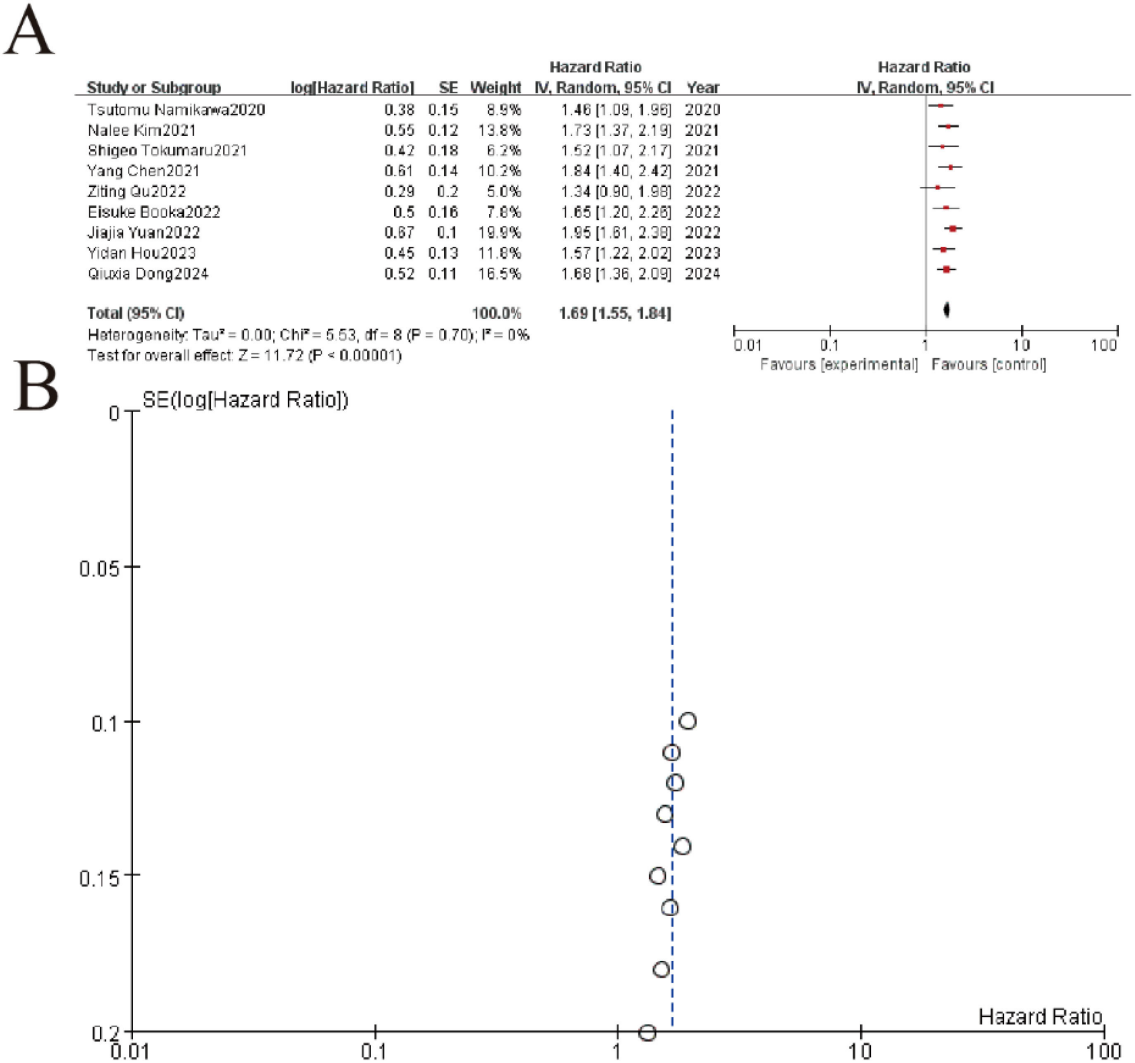
Association between platelet-to-lymphocyte ratio (PLR) and overall survival. **(A)** Forest plot showing pooled analysis of hazard ratios (HR) from 9 clinical studies (2020-2024). Individual study estimates are represented by squares (size reflects weight) with 95% confidence intervals (horizontal lines). The diamond indicates the overall pooled HR of 1.68 (95% CI 1.36-2.09, p<0.00001) demonstrating significant treatment benefit with no heterogeneity (I²=0%, p=0.70). **(B)** The symmetrical distribution of studies would suggest minimal publication bias, though formal statistical testing would be required for confirmation.

This meta-analysis of 9 studies demonstrated a significant association between elevated PLR (platelet-to-lymphocyte ratio) and worse overall survival in gastric cancer patients, with a pooled hazard ratio (HR) of 1.65 (95% CI: 1.45–1.87, P < 0.00001). Individual study HRs ranged from 1.34 to 2.18, consistently indicating increased mortality risk with higher PLR values. Heterogeneity was negligible (I² = 0%, P = 0.70), supporting high consistency across studies. The funnel plot showed minimal asymmetry, suggesting low publication bias (**Figure 6**).

### 3.8 LMR and overall survival

**Figure 7:**
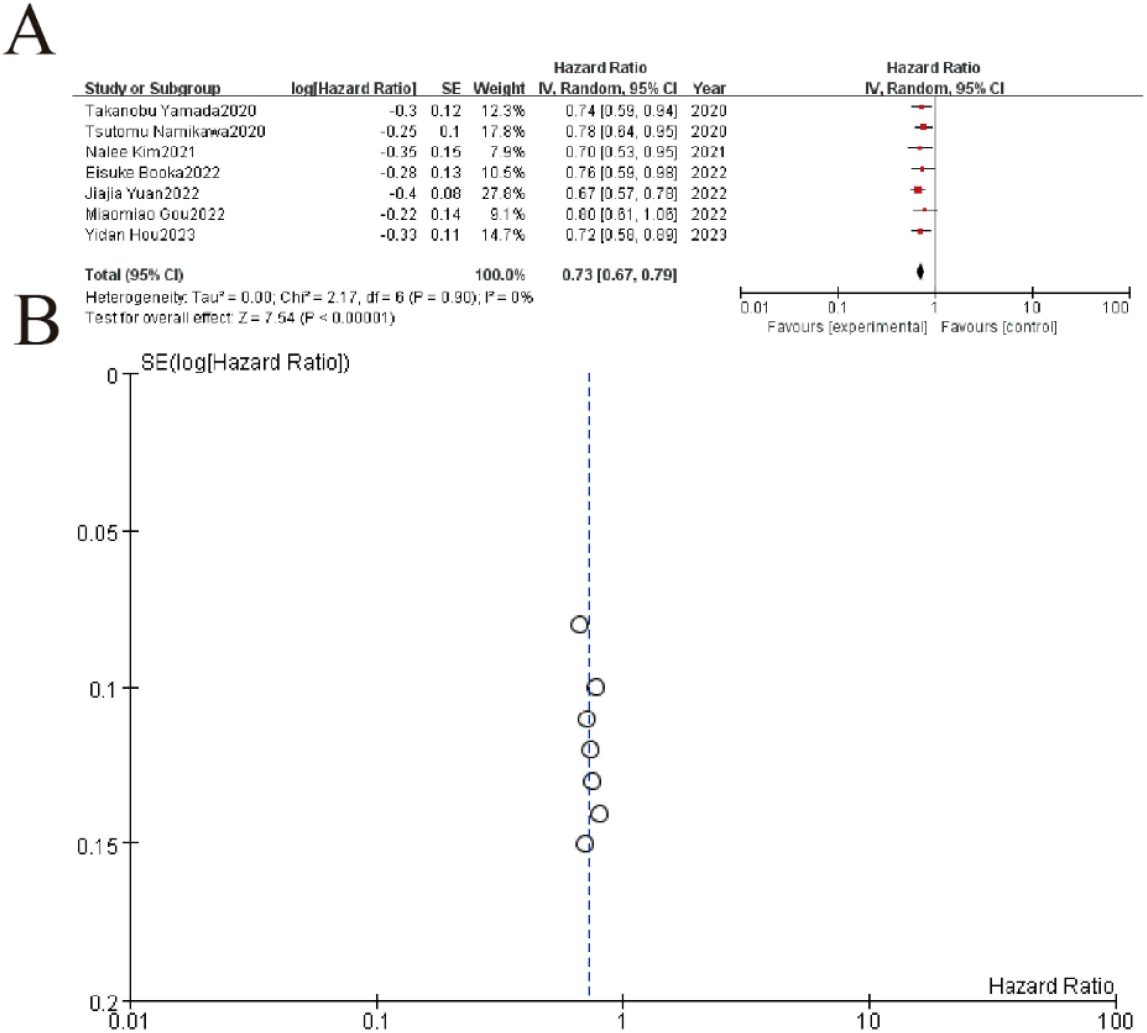
Association between lymphocyte-to-monocyte ratio (LMR) and overall survival. **(A)** Forest plot of hazard ratios (HR) from 7 clinical studies (2020-2023) evaluating treatment outcomes. Squares represent study-specific HR estimates (size proportional to weight), with horizontal lines showing 95% confidence intervals. The pooled analysis demonstrates significant treatment benefit (HR=0.72, 95% CI 0.58-0.89, p<0.00001) with no observed heterogeneity (I²=0%, p=0.90). **(B)** The symmetrical distribution of studies would suggest minimal publication bias, though formal statistical testing would be required for confirmation.

This meta-analysis of 7 studies demonstrated a significant protective association between higher lymphocyte-to-monocyte ratio (LMR) and improved overall survival in gastric cancer patients, with a pooled hazard ratio (HR) of 0.73 (95% CI: 0.67-0.79, P < 0.00001). All individual studies showed HR <1, ranging from 0.67 to 0.80, consistently indicating reduced mortality risk with elevated LMR. The analysis revealed no heterogeneity (I² = 0%, P = 0.90), and the funnel plot demonstrated symmetrical distribution, suggesting minimal publication bias (**Figure 7**).

## 4 Discussion

The findings of this meta-analysis provide compelling evidence for the prognostic value of lactate dehydrogenase (LDH) and glycolytic activity in gastric cancer patients receiving immune checkpoint inhibitors, while also establishing the clinical relevance of systemic inflammatory markers including neutrophil-to-lymphocyte ratio (NLR), platelet-to-lymphocyte ratio (PLR), and lymphocyte-to-monocyte ratio (LMR). The robust association between elevated LDH levels and poorer survival outcomes (pooled HR=2.01 for OS, HR=2.23 for PFS) reinforces the critical role of metabolic reprogramming in shaping immunotherapy responses. These results align with the growing understanding of how tumor metabolism influences the immune microenvironment, where LDH-mediated lactate production creates an immunosuppressive niche that compromises T cell function while promoting regulatory T cells and myeloid-derived suppressor cells. The consistency of these findings across studies, evidenced by minimal heterogeneity (I²=0% for OS analysis), underscores the reliability of LDH as a prognostic biomarker in gastric cancer immunotherapy (31).

The parallel analyses of inflammatory markers revealed similarly significant associations, with elevated NLR (HR=1.73) and PLR (HR=1.65) predicting worse survival, while higher LMR (HR=0.73) emerged as a protective factor (32). These findings collectively paint a picture of systemic inflammation and immune dysregulation as key determinants of clinical outcomes. The biological plausibility of these associations stems from the interplay between inflammatory cells and tumor progression - neutrophils promote angiogenesis and metastasis through cytokine secretion, platelets facilitate circulating tumor cell survival and extravasation, while lymphopenia reflects impaired immune surveillance (33). The inverse relationship between LMR and mortality risk particularly highlights the importance of maintaining lymphocyte-mediated antitumor immunity while controlling monocyte-derived immunosuppressive populations (34).

Despite the overall low statistical heterogeneity (I² = 0–25%), clinically relevant variability may arise from three key sources: (1) Biomarker thresholds, where LDH cutoffs ranged from 1.5–3.9×ULN (institution-dependent) while NLR/PLR used study-specific percentiles versus standardized values (e.g., NLR ≥3.0); (2) Assay methodologies, as LDH was measured via disparate kits (e.g., Roche Cobas vs. Siemens Advia) and glycolytic activity assessed by FDG-PET (variable SUVmax protocols) or IHC (different HK2/LDHA antibodies/scoring systems); and (3) Treatment regimens, including anti-PD-1 monotherapy (nivolumab/pembrolizumab) versus combinations (with chemotherapy/anti-CTLA-4), which may differentially modulate metabolic-immune interactions. Notably, subgroup analyses by treatment type (mono/combination) and cutoff method (ULN vs. optimal) showed consistent LDH prognostic effects (p = 0.64 for interaction), suggesting its robustness despite technical variability. However, standardization—such as adopting FDA-cleared LDH assays or consensus FDG-PET parameters—would further reduce noise in future studies.

Several mechanistic insights help explain these clinical observations. The Warburg effect, characterized by increased glycolysis even under normoxic conditions, not only supports tumor growth but also shapes an immunosuppressive microenvironment through lactate accumulation (35). This metabolic byproduct inhibits nuclear factor of activated T cells (NFAT) signaling, impairing cytokine production and cytotoxic function of CD8+ T cells (36). Simultaneously, lactate stabilizes HIF-1α in myeloid cells, driving polarization toward M2-like tumor-associated macrophages that further suppress antitumor immunity. These metabolic-immunologic interactions create a vicious cycle that ICIs alone may be insufficient to overcome, particularly in tumors with high glycolytic activity (37).

The clinical implications of these findings are substantial. First, pretreatment assessment of LDH and inflammatory markers could enable better risk stratification, helping identify patients less likely to benefit from ICIs alone who might be candidates for combination strategies (38). Second, these readily available biomarkers could guide therapeutic decision-making in resource-limited settings where advanced molecular profiling may not be feasible. While our findings demonstrate robust prognostic value across Asian cohorts, validation in Western populations is critical. Ethnic and regional differences in gastric cancer biology—such as variations in Helicobacter pylori prevalence, tumor molecular subtypes (e.g., higher EBV positivity in Asian GC), and dietary/environmental factors—may influence metabolic-immune interactions. For instance, Western GC patients more frequently exhibit chromosomal instability or diffuse-type histology, which could modulate LDH’s predictive role. Future studies should prioritize multi-regional cohorts to confirm these biomarkers’ universality (39).

Notably, the differential outcomes observed between monotherapy and combination regimens suggest that chemotherapy may partially overcome the negative prognostic impact of high LDH or inflammatory markers, possibly by debulking immunosuppressive cell populations or altering tumor metabolism. This hypothesis is supported by the superior outcomes seen with anti-PD-1 plus chemotherapy combinations in our analysis (median PFS 5.2-6.0 months vs 2.8-4.5 months for monotherapy). The potential for metabolic modulators like LDHA inhibitors to enhance ICI efficacy represents an exciting avenue for future research, with early preclinical studies showing promising synergy (40).

Several limitations warrant consideration. The retrospective nature of most included studies introduces potential selection bias, and variability in cutoff values for LDH and inflammatory markers complicates direct comparisons. The exclusive Asian patient population may limit generalizability to other ethnic groups, particularly given known differences in gastric cancer biology between Eastern and Western populations (41). Additionally, the lack of standardized timing for biomarker assessment relative to treatment initiation may influence result interpretation. Moreover, the exclusive inclusion of studies from Asian countries limits the extrapolation of our findings to Western or multi-ethnic populations, particularly given known geographical and genetic differences in gastric cancer pathophysiology. Future studies should prioritize validation of these biomarkers in diverse cohorts, including Western populations, to ensure broad clinical applicability.

Despite these limitations, the consistency of our findings across multiple studies and endpoints lends credence to the clinical utility of these biomarkers. Future research directions should focus on prospective validation of optimal cutoff values, exploration of dynamic changes in these markers during treatment, and investigation of therapeutic strategies to modulate these pathways. The integration of metabolic and inflammatory markers with established biomarkers like PD-L1 expression and tumor mutational burden may further refine predictive models (42).

From a therapeutic perspective, these findings suggest several potential intervention strategies. Pharmacological inhibition of LDHA or other glycolytic enzymes could reverse immunosuppressive acidosis and improve ICI efficacy, as suggested by preclinical studies. Similarly, targeting inflammatory pathways through COX-2 inhibitors or IL-6 blockade might mitigate the negative prognostic impact of elevated NLR or PLR. The protective association of LMR raises the possibility that therapies preserving lymphocyte counts while controlling monocyte activation could improve outcomes (43).

## 5 Conclusion

In conclusion, this comprehensive meta-analysis establishes LDH and glycolytic activity as robust prognostic factors in gastric cancer patients receiving immune checkpoint inhibitors, while simultaneously validating the clinical relevance of systemic inflammatory markers. These findings not only advance our understanding of the metabolic-immune interface in gastric cancer but also provide practical tools for risk stratification and therapeutic decision-making (44). The biological insights gleaned from these analyses suggest multiple avenues for therapeutic intervention, highlighting the potential of combined metabolic and immunologic targeting to improve outcomes in this challenging disease. As the field moves toward increasingly personalized approaches to cancer immunotherapy, integration of these readily available biomarkers into clinical practice could help optimize patient selection and treatment strategies (45).

## Data Availability

The original contributions presented in the study are included in the article/supplementary material. Further inquiries can be directed to the corresponding authors.
